# Fractures are increased and bisphosphonate use decreased in individuals with insulin-dependent diabetes: a 10 year cohort study

**DOI:** 10.1186/1471-2474-15-201

**Published:** 2014-06-11

**Authors:** Lisa-Ann Fraser, Alexandra Papaioannou, Jonathan D Adachi, Jinhui Ma, Lehana Thabane

**Affiliations:** 1 Department of Medicine, University of Western Ontario, London, Ontario, Canada; 2 Department of Epidemiology and Biostatistics, McMaster University, Hamilton, Ontario, Canada; 3 Department of Medicine, McMaster University, Hamilton, Ontario, Canada; 4 Division of Endocrinology and Metabolism, St. Joseph’s Hospital, 268 Grosvenor Street, London N6A 4 V2 Ontario, Canada

**Keywords:** Fracture, Diabetes, Insulin, Care gap, Treatment, Osteoporosis

## Abstract

**Background:**

Individuals with diabetes have been found previously to be at increased risk of non-traumatic fracture. However, it is unclear if these individuals are being identified and treated for osteoporosis.

**Methods:**

7753 Canadians over 50 years of age were followed prospectively for 10 years. 606/7753 (7.8%) of had diabetes; 98 were insulin-dependent and 508 were not. Using a cox proportional hazards model, we assessed the association between diabetes status and incident non-traumatic fracture. Using logistic regression we identified factors associated with bisphosphonate use over the 10 year period of study.

**Results:**

Mean (SD) age of participants was 66.7(9.4) years and 72% were female. Those with diabetes had higher BMD T-scores at baseline, with a mean (SD) femoral neck T-Score of -0.97 (1.06), compared to -1.24 (0.99) in the general cohort. The adjusted hazard ratio (HR) for incident non-traumatic fracture in individuals with insulin-dependent diabetes over the 10 year study period was 2.50 (95% confidence interval [CI] 1.60, 3.90; p < 0.001). Despite this increased fracture rate, individuals with diabetes (insulin-dependent or non-insulin-dependent) were less likely to be on bisphosphonate therapy at any point over 10 years of prospective follow up compared to other CaMos subjects (odds ratio [OR]: 0.59; 95% CI 0.46-0.75, p < 0.001).

**Conclusions:**

Despite the increased risk of non-traumatic fracture associated with insulin-dependent diabetes, we that found individuals with diabetes are less likely to be treated with a bisphosphonate than those without diabetes. These findings point to a possible care gap in the treatment of non-traumatic fractures in individuals with diabetes in Canada.

## Background

Osteoporosis is a common condition in Canada, affecting 21% of women and 5% of men over the age of 50 years [1]. Multiple observational studies have been performed around the world showing that diabetes (both type 1 and type 2) is associated with an increased risk of non-traumatic fracture. Non-traumatic, or “fragility” fractures are fractures that occur with minimal trauma, such as a fall from standing height or less [2,3]. A prior fracture is an important risk factor for having future fractures [4]. Recent meta-analyses indicate an increased relative risk for hip fracture in type 2 diabetics of 1.4 to 1.7 and in type 1 diabetics of 6.3 to 6.9 [5,6]. Other skeletal sites have also been found to be at increased risk of fracture in the diabetic population [7,8].

In the only Canada-wide study performed to-date, Hanley et al. examined prevalent vertebral deformities, as measured by spinal radiographs, and found no significant increase in individuals with type 1 diabetes (OR: 1.24; 95% CI 0.68,2.51) or type 2 diabetes (OR: 0.91; 0.67,1.25) [9]. However, no study to-date has prospectively examined non-traumatic clinical fractures (both vertebral and non-vertebral) in the diabetic population in Canada. Similarly, to the best of our knowledge, there are no published studies looking at osteoporosis treatment rates amongst the diabetic population. Given the osteoporosis treatment “care gap” that has been identified in the general population [10,11], we hypothesize that an even greater care gap exists within the diabetic population. The primary objective of this study was to evaluate the relationship between diabetes and incident non-traumatic fracture, with the secondary aim of evaluating associated bisphosphonate treatment over a 10 year period, in a large population-based longitudinal cohort study, including men and women with insulin-dependent and non-insulin-dependent diabetes in Canada.

## Methods

The Canadian Multicenter Osteoporosis Study (CaMos) is an on-going population-based cohort study looking at osteoporosis and fracture risk in community dwelling Canadians. Baseline questionnaires were completed in 1995–1997. The institutional review boards of all sites participating in CaMos approved the study and informed consent was obtained from all participants. The study has been described in detail elsewhere [12], but areas relevant to this study are summarized below. Data from years 0 through 10 were used for this study and all women and men aged 50 years or older were included in the analyses.

Review Boards that approved the CaMos study:

McGill University Health Centres-Montreal General Hospital Research Ethics Committee.

Conjoint Health Research Ethics Board of the Faculty of Medicine, University of Calgary.

St. Joseph’s Healthcare, McMaster University Research Ethics Board.

Queen’s University Research Ethics Board.

Memorial University of Newfoundland, Human Investigations Committee.

University of Saskatchewan Advisory Committee on Ethics in Human Experimentation.

St. Michael’s Hospital Research Ethics Board (Toronto).

Capital Health Research Ethics Board (Halifax).

The University of British Columbia Clinical Research Ethics Board.

Centre hospitalier de l’Université Laval Comite d’Ethique de la Recherche Clinique.

### Study participants

Participants were recruited from within 50-kilometers of one of 9 study centers across Canada (St. John’s, Halifax, Quebec City, Toronto, Hamilton, Kingston, Saskatoon, Calgary and Vancouver). 9,423 individuals (6,539 females and 2,884 males) aged 25 years and older, representing an age-stratified-, sex-, and region-specific sample, were identified from lists of random telephone numbers over an 18 month period.

### Data collection

An interviewer-administered questionnaire was performed at baseline, and at years 3, 5 and 10 of the study. Diabetes status was captured in the baseline questionnaire where participants were asked if they had insulin dependent diabetes mellitus (IDDM) or non-insulin dependent diabetes mellitus (NIDDM). All past medical history was by patient report. At baseline, year 5, and 10, bone mineral density (BMD) testing was performed. At years 1, 2, 4, 6, 7, 8 and 9, a two-page questionnaire was mailed to participants which included questions about bone-related medications. All participant medications were documented in detail during the interviewer-administered questionnaires.

### Bone mineral density and fractures

BMD of the hip and lumbar spine (L1-L4) were measured by dual-energy X-ray absorptiometry (DXA) using Hologic QDR 1000, 2000, 4500 or Lunar DPX machines. Densitometers were calibrated daily, and quality assurance was performed following a standard daily and weekly schedule. Initially, cross-calibration of the machines was performed at the nine centers using a European Spine Phantom. After this, the Bone Fide phantom was performed at baseline and in the year of every examination. Reports indicating bone density (g/cm2), and T-scores were sent to each participant, a physician named by the participant, or both depending on the centre [13]. All clinically recognized non-traumatic fractures were included in the analysis (hip fractures, clinical vertebral fractures, non-vertebral fractures). Any fracture associated with trauma or described as a fall from more than standing height was excluded. At baseline, previous fractures were obtained by self-report, but subsequent fractures were reported by patients and confirmed by medical or radiographic reports.

### Statistical analysis

All analyses were restricted to CaMos participants ≥ 50 years of age. Baseline characteristics were described using mean (standard deviation) for continuous variables and count (percentage) for nominal variables. The primary objective of determining the association between diabetes (insulin-dependent and non-insulin dependent) and incident non-traumatic fracture was examined using a Cox Proportional Hazards regression model, adjusting for age, gender, baseline femoral neck T-score, history of previous non-traumatic fracture, body mass index (BMI), bisphosphonate use and past use of corticosteroids. Age was divided into 10-year categories (50–59, 60–69, 70–79, ≥80). Age 50–59 was used as the reference category in the analyses. Diabetes was defined as either insulin-dependent diabetes mellitus (IDDM) or non-insulin-dependent diabetes mellitus (NIDDM). BMD was divided into femoral neck T-scores of ≥ -1.0, <-1.0 to > -2.5, and ≤ -2.5. BMI was divided into categories including: <18.5, ≥ 18.5 to <25, ≥25 to <30, ≥30 to <35, and ≥ 35. Bisphosphonate use (any of: alendronate, clodronate, etidronate, pamidronate, risedronate, and zoledronic acid) at any time point over the 10 year study was included. Past corticosteroid use included ever daily use of IV or oral corticosteroids for at least one month. All independent variables adjusted for in the model were selected because they are risk factors that have previously been identified as having important associations with fracture risk. Proportional hazard assumptions were tested using Schoenfeld residual test. Logistic regression modeling was used to determine factors associated with bisphosphonate use. “Ever bisphosphonate use” (ie. patient reported being on a bisphosphonate at any time point during the 10 years studied) was the dependent variable and diabetes status, age, gender, femoral neck T-score, rheumatoid arthritis, family history of osteoporosis, and history of non-traumatic fracture at baseline, were covariates. Sensitivity analysis using multiple imputation [14] was performed for missing data to assess the robustness of the model assuming the data are missing at random. The criterion for statistical significance was set at alpha = 0.05. We used the Hosmer and Lemeshow test to assess goodness-of-fit of logistic regression. Cox proportional hazards modelling and Schoenfeld residual testing were performed using SAS 9.2 (Cary, NC). Descriptive analysis, logistic regression modeling, and multiple imputation were performed using IBM SPSS version 19 (Ireland).

## Results

Characteristics of the CaMos population over 50 years of age (n = 7753) and of participants with insulin-dependent diabetes (n = 98) and non-insulin-dependent diabetes (n = 508) have been described previously and are shown in Table 1[15]. Most baseline characteristics were similar between all groups. The diabetic groups however, had higher BMD values than the general CaMos population (Table 2). Despite these higher values, individuals with diabetes were more likely to have had a non-traumatic fracture at baseline (39% of IDDM and 29% of NIDDM compared to 27% of the general CaMos population). Individuals with IDDM reported a longer duration of diabetes, 15 compared to 10 years in those with NIDDM.

**Table 1:** **Baseline characteristics of all CaMos participants over 50 years and of participants with diabetes**[15]

	**All CaMos participants >50 yrs (n = 7753)**	**Insulin-dependent diabetes (n = 98)**	**Non-insulin-dependent diabetes (n = 508)**
**Age (years); mean (SD)**	66.7 (9.4)	68.0 (9.0)	69.4 (8.8)
**Gender (female)**	71.8%	64.3%	65.7%
**Femoral neck BMD T-score; mean (SD)**	-1.24 (0.99)	-0.97 (1.17)	-0.97 (1.04)
**Caucasian**	95.5%	93.9%	94.3%
**Fracture at baseline (n;%)**	2133 (27.5)	38 (38.8)	146 (28.7)
**BMI; mean (SD)**	27.1 (4.78)	29.73 (5.45)	29.06 (5.24)
**Years since diagnosis of diabetes; mean;(SD)**	N/A	15.4 (11.28)	9.64 (9.60)
Cigarette use;* n (%)	4163 (53.7)	54 (55.1)	280 (55.1)
**Corticosteroid use;**^**†**^**n(%)**	415 (4.4)	12 (12.24)	33 (6.50)
**Alcohol use (per wk); mean (SD)**	2.88 (5.85)	1.97 (5.45)	2.27 (6.79)

*ever use daily for > 6 months.

^†^oral or IV, ever use daily for > 1 month.

SD: standard deviation, BMD: bone mineral density, BMI: body mass index.

**Table 2:** Baseline bone mineral density values, and proportion of individuals on bisphosphonates at baseline, in CaMos participants with and without diabetes, by BMD Category

**Femoral neck T-score**	**No diabetes**	**BP use**	**Insulin-dependent diabetes**	**BP use**	**Non-insulin-dependent diabetes**	**BP use**
**> - 1.0**	44.4%	0.2%	44.9%	0.0%	51.5%	0.0%
**-1.0 to -2.5**	48.3%	1.9%	48.7%	2.6%	43.2%	3.2%
**<-2.5**	7.3%	6.5%	6.4%	20%	5.3%	8.7%

*BP*: Bisphosphonate.

**Figure 1:**
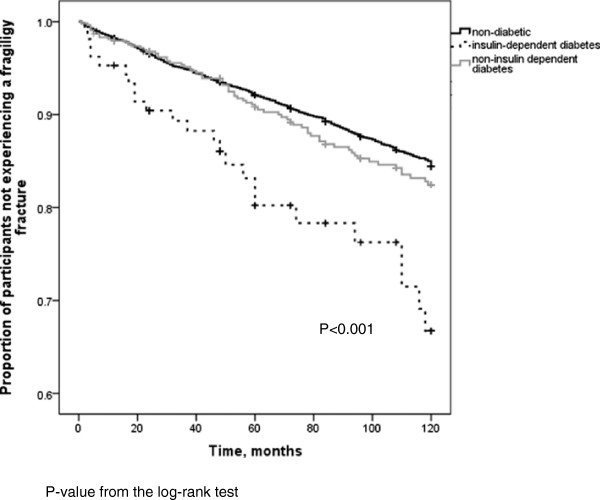
Kaplan-Meier curve showing unadjusted fragility fracture free survival by diabetes status.

**Figure 2:**
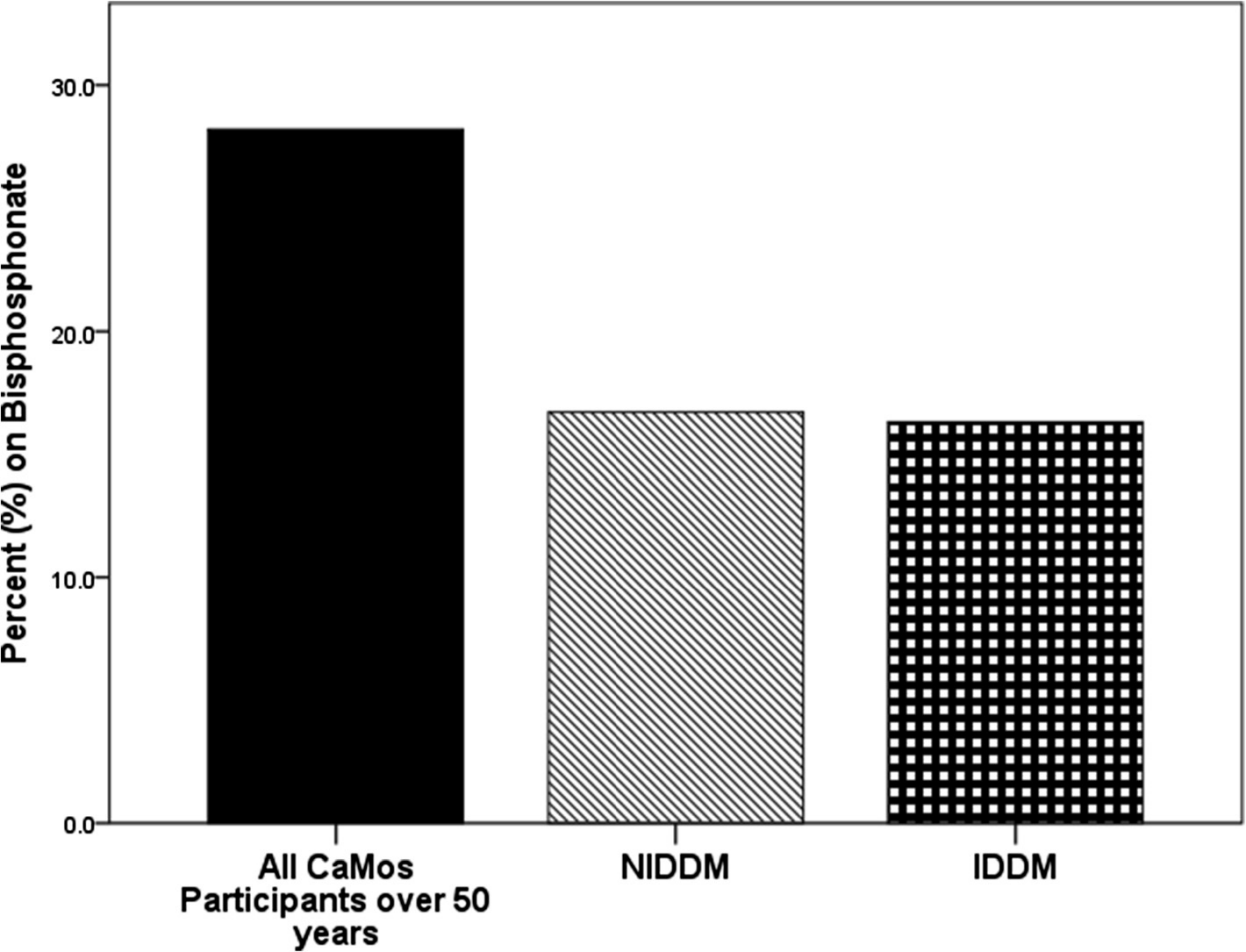
CaMos participants on bisphosphonate therapy with at any time during a 10 year follow-up period.

Incident fractures occurred in 1098 non-diabetic individuals, with the site of first incident fracture being the hip in 142 individuals and vertebrae in 117 individuals. Among those with diabetes, 73 individuals sustained one or more incident fractures over the 10 year period, 21 individuals with IDDM and 52 with NIDDM. Of these, hip was the site of first incident fracture in 2 individuals with IDDM and 10 individuals with NIDDM. Clinical vertebral fracture accounted for first incident fracture in 3 participants with IDDM and 5 with NIDDM. The adjusted hazard ratio (HR) for incident non-traumatic fracture in individuals with insulin-dependent diabetes over the 10 year study period was 2.5 (95% CI 1.60, 3.90; p < 0.001) (Figure 1). Other variables associated with an increased risk of fracture included: female gender, history of prior fragility fracture, increased BMI, older age, lower femoral neck T-score, corticosteroid use and bisphosphonate use (Table 3). Non-insulin-dependent diabetes was not found to be associated with increased fracture risk, HR: 1.02, 95%CI 0.77-1.35, p = 0.869. Statistical testing showed the proportional hazards assumption was not violated. Individuals with diabetes were less likely to be on bisphosphonate therapy during the 10 year study period compared to other CaMos subjects (OR: 0.68; 95% CI 0.50, 0.93; p = 0.016). In participants without diabetes who suffered an incident fracture, 536 (48.8%) reported bisphosphonate use during CaMOs, whereas 6 individuals (28.6%) with IDDM who sustained an incident fracture and 16 individuals (30.8%) with NIDDM and incident fracture reported use. After multiple imputation was performed for the variables: rheumatoid arthritis, family history of osteoporosis and femoral neck BMD; the relationship strengthened (OR: 0.59; 95% CI 0.46, 0.75; p < 0.001) (Table 4). Other variables associated with decreased likelihood of bisphosphonate use were older age and increased femoral neck T-score. Variables associated with increased bisphosphonate use included: rheumatoid arthritis, family history of osteoporosis, female gender, and history of non-traumatic fracture. When insulin-dependent and non-insulin-dependent diabetes and bisphosphonate use was examined, the results were not statistically significant prior to multiple imputation. However, after multiple imputation for variables listed above, non-insulin-dependent diabetes was found to be associated with decreased use of bisphosphonates (OR: 0.59; 95% CI 0.45, 0.78, p < 0.001), as was insulin-dependent diabetes (OR: 0.53; 95% CI 0.29, 0.95; p = 0.034) as shown in Figure 2. When the diabetic subgroup was examined, similar risk factors (steroid use, age, female gender, lower BMD, and past fracture) were associated with bisphosphonate use.

**Table 3:** Variables associated with risk of incident non-traumatic fracture over 10 years (multivariate analysis)

**Variable**	**Hazard ratio**	**95% Confidence interval**	**Significance (p-value)**
**Diabetes status**			
No diabetes	reference		
Non-insulin-dependent diabetes mellitus	1.02	0.77-1.35	0.864
Insulin-dependent diabetes mellitus	2.50	1.60-3.90	<0.001
**Gender**			
Male	reference		
Female	1.54	1.30-1.82	<0.001
**Age (years)**			
50-59	reference		
60-69	0.95	0.80-1.13	0.551
70-79	1.40	1.17-1.67	<0.001
≥ 80	1.69	1.30-2.19	<0.001
**Body mass index**			
18.5 ≤ BMI <25	reference		
BMI < 18.5	0.82	0.50-1.37	0.451
25 ≤ BMI <30	1.09	0.94-1.26	0.235
30 ≤ BMI < 35	1.28	1.06-1.54	0.011
BMI ≥ 35	1.35	1.02-1.80	0.037
**Femoral neck BMD T-score (per SD increase)**	0.69	0.64-0.76	<0.001
BMD ≥ -1.0	reference		
-2.5 < BMD < -1.0	1.59	1.35-1.88	<0.001
BMD ≤ -2.5	2.56	2.04-3.21	<0.001
**Previous history of fracture**			
No	reference		
Yes	1.58	1.39-1.79	<0.001
Corticosteroid use^*^			
No	reference		
Yes	1.35	1.07-1.69	0.010
**Bisphosphonate use (ever use over the 10 year study)**			
No	reference		
Yes	1.56	1.36-1.79	<0.001

*ever use daily for > 6 months.

**Table 4:** Variables associated with bisphosphonate use over the 10 year study period (after multiple imputation)

**Variable**	**Odds ratio**	**95% Confidence interval**	**P-value**
**Diabetes**	0.59	0.46-0.75	<0.001
**Previous non-traumatic fracture**	1.20	1.06-1.36	0.004
**Age (per year)**	0.99	0.98-0.99	<0.001
**Femoral neck T-score (per SD increase)**	0.39	0.36-0.42	<0.001
**Rheumatoid arthritis**	1.28	1.03-1.59	0.029
**Family history of osteoporosis**	1.24	1.06-1.44	0.008
**Female gender**	3.00	2.59-3.47	<0.001

## Discussion

In our analysis, we found that Canadians with insulin-dependent diabetes are more likely to sustain an incident non-traumatic fracture than other Canadians over 50 years of age. Despite this increased risk, Canadian diabetics in CaMos (both insulin-dependent and non-insulin-dependent) were 42% less likely than non-diabetics to be treated with a bisphosphonate (first line therapy for prevention and treatment of fragility fractures) over the 10 year study period; indicating a significant care-gap in skeletal care amongst Canadians with diabetes.

The diabetic groups in CaMos had higher BMD values than the general CaMos population. This finding is similar to other studies which have shown higher BMD values in type 2 diabetics, likely on the basis of elevated BMI which is a risk factor for developing type 2 diabetes [6,16,17]. This difference in BMI was also evident in the CaMos population (BMI of 27 vs. 29 in diabetic participants). The higher bone density values however did not protect the participants with diabetes from fracturing.

At study baseline, we found an increased history of non-traumatic fractures amongst all diabetics in CaMos, this is in-keeping with previous literature showing this population to be at elevated fracture risk [5,6]. Looking prospectively, we found an increased risk of incident non-traumatic fracture in diabetics on insulin, but we did not demonstrate an increased risk in diabetics not on insulin. In Canada, information about fracture risk in those with diabetes has not been abundant. The only study to-date, also performed in the CaMos population, focused on prevalent vertebral deformities by radiograph and showed no increased risk in type 1 or type 2 diabetes [9]. Our study shows an increase in clinical non-traumatic fractures, at any body site, in individuals with insulin-dependent diabetes at a national level. These findings support previous, province-wide studies, showing increased risk of hip fracture, as well as other osteoporotic fractures, in type 2 diabetics [18,19]. Similar to our findings, studies done in other countries have shown insulin use further increases the risk of fragility fractures in individuals with diabetes, perhaps by acting as a marker of disease severity [20-22]. Other explanations for the association between insulin use and fracture include increased duration of type 2 diabetes, and the inclusion of individuals with type 1 diabetes [20]. Glycemic control was not assessed in CaMos. It is not clear if our finding of no increased risk of fracture in non-insulin-dependent diabetics in CaMos relates to a lack of statistical power needed to demonstrate a more subtle risk increase in this population, or if this indeed signifies no increased risk in this group. Given that we found those with NIDDM had an increased prevalence of fragility fractures at our study baseline (compared to the non-diabetic population) and that previous studies have consistently reported an increased fracture risk in this population, we suspect a power issue is an important contributor to our findings [5,6]. Our study was not designed to identify the cause of increased fracture risk in diabetics; multiple different pathological mechanisms have been reported in the literature in the past [23,24]. We did not have access to measures of glycemic control, and therefore did not examine its influence on fracture risk.

We found that, despite having increased rates of non-traumatic fractures, individuals with diabetes were less likely to receive treatment with a bisphosphonate than other CaMos participants. To our knowledge, this care-gap has not been identified in the diabetic population previously. Within the general population however, it is well known that a large care gap exists, with many patients not being diagnosed or treated for their osteoporosis. This care-gap has been documented both in Canada and internationally [25,26]. Within the general CaMos population there have been reports of a care-gap in men and women with fragility fractures; approximately half of women experiencing a new fragility fracture were found to not be treated with a bone-specific medication [10] and over 90% of men were untreated [11]. Considering osteoporosis treatment rates within the CaMos population are low, our findings of an even larger care-gap amongst those with diabetes within CaMos suggests a dire situation for these patients. Moreover, we found that those with IDDM were the most likely to have had a fracture in the past and were at higher risk of having an incident fracture over the 10 year study period, yet were the least likely to receive treatment with a bisphosphonate over the 10 years of the study. Although large randomized control trials proving the efficacy of bisphosphonate therapy for fracture prevention in the setting of diabetes are lacking, a large observational study and a sub-group analysis of diabetic participants in the Fracture Intervention Trial have found that the improvement in BMD and the decrease in fracture risk associated with bisphosphonate therapy is not altered by the diagnosis of diabetes. Similarly, the tolerability of Alendronate has been found to be no different in diabetic vs. non-diabetic women [27,28]. There are no accepted theories as to why a diabetes-osteoporosis care gap exists above the level of the baseline care-gap in the general population. One possibility may be the higher BMD values typically found in individuals with type 2 diabetes. Traditionally, most clinicians have used BMD scores to diagnose and make treatment decisions around osteoporosis. This bias is reflected in past osteoporosis treatment guidelines [29]. Although newer guidelines focus more on fracture risk assessment, and less on BMD alone, many clinicians still rely heavily on BMD when making treatment decisions. It is therefore possible that the normal or elevated BMD values that are typically seen in type 2 diabetics make clinicians less likely to suspect, or treat, osteoporosis. This highlights the need for education and knowledge dissemination to diabetic practitioners about the link between diabetes and fracture.

This study has several strengths including the large population-based sample, prospective design allowing 10 years of follow-up, inclusion of both men and women, detailed fracture data, and the ability to differentiate between insulin-dependent and non-insulin-dependent diabetes. There are however several limitations to this study. All the CaMos questionnaires depended on patient reporting (subject to recall bias and misunderstanding), therefore the incidence of certain classically underdiagnosed conditions (such as hyperglycemia) is likely underestimated. Similarly, individuals who were not diabetic at study baseline but became so during the course of the study, or diabetics who became insulin-dependent later over the 10 year study, were not captured. However, bias from undiagnosed diabetes (or insulin use) would be expected to decrease the effect sizes found. All fractures included in the analyses were clinical fractures; morphometric vertebral fractures were not included. The CaMos population is mostly of Caucasian ethnicity and therefore results cannot be extrapolated to other race groups. Thiazolidinediones, implicated in increasing fracture risk [30,31], were not included in our analysis because no CaMos participants were on one of these medications at study baseline.

We had originally hoped to perform this study with diabetes broken down into type 1 and type 2, rather than IDDM and NIDDM, as the pathophysiology and epidemiology of skeletal fragility is different in type 1 vs. type 2 diabetes [23,24]. However, upon examination of the self-reported age of diagnosis of “type 1 diabetes” in the CaMos baseline questionnaire we found that only 10 individuals (9.6% of self-reported insulin-dependent diabetics) were under the age of 30 years when they were diagnosed with diabetes. This led us to suspect that this group consists mostly of type 2 diabetics who are insulin-dependent (relative insulin deficiency) rather than type 1 diabetics (absolute insulin deficiency). Supporting this is the high baseline BMI and BMD values found in both diabetic groups, which are more typical of type 2 diabetes than type 1 [6]. In the past, diabetes was often divided into “insulin-dependent diabetes” and “insulin-independent” diabetes; terms that are not generally used in the medical community today [32]. However, this often leads to confusion amongst people with diabetes who obtained their diabetes education and diagnosis when these terms were commonplace. We therefore decided to report our results as IDDM and NIDDM, as we questioned the accuracy of the type1/type 2 classification.

## Conclusions

In summary, we found that Canadians with insulin-dependent diabetes mellitus were more likely than non-diabetics to sustain a non-traumatic fracture over a 10 year period. Despite this increased risk, diabetics are less likely to receive fracture prevention therapy with a bisphosphonate. Clinicians that treat individuals with diabetes (especially those treated with insulin) should be taught to incorporate fracture prevention into the current list of interventions they offer to diabetic patients. Future studies are needed to clarify if individuals with non-insulin-dependent diabetes are at increased fracture risk, to validate fracture risk assessment tools within the diabetic population and to test fracture prevention strategies and therapies specifically in the diabetic population.

## Competing interests

LAF: speakers bureau for Amgen; AP: consultant/speaker for: Amgen, Aventis, Eli Lilly, Merck Frosst, Novartis, Procter & Gamble, Servier, and Wyeth-Ayerst; conducted clinical trials for: Eli Lilly, Merck Frosst, Novartis, Procter & Gamble, and Sanofi-Aventis; and received unrestricted grants from: Amgen, Eli Lilly, Merck Frosst, Procter & Gamble, and Sanofi-Aventis; JDA: research support and consultant: Amgen, Astra Zeneca, Eli Lilly, GlaxoSmithKline, Merck, Novartis, Nycomed, Pfizer, Procter and Gamble, Roche, Sanofi Aventis, Servier, Warner Chilcott, Wyeth; JM: no disclosures; LT: no disclosures.

## Authors’ contributions

LAF was involved with the concept and design of this study as well as the analysis and interpretation of data. She also wrote the manuscript. AP and JDA participated in the study design, data acquisition, and revising of the manuscript. JM was involved with data analysis and revising the manuscript. LT participated in the study design, data analysis and revision of the manuscript. All authors read and approved the final manuscript.

## Pre-publication history

The pre-publication history for this paper can be accessed here:

http://www.biomedcentral.com/1471-2474/15/201/prepub
